# Alterations in peripheral CD4^+^ T cell subsets and their association with metabolic markers in obese children

**DOI:** 10.3389/fendo.2025.1635379

**Published:** 2025-09-22

**Authors:** Reiko Saito, Jun Hirakawa, Mami Kuwamura, Takayuki Hoshina

**Affiliations:** ^1^ Department of Pediatrics, School of Medicine, University of Occupational and Environmental Health, Kitakyushu, Japan; ^2^ General Medical Department, Fukuoka Children’s Hospital, Fukuoka, Japan

**Keywords:** childhood obesity, CD4+ T cells, immune dysregulation, insulin resistance, lymphocyte subpopulations, metabolic dysfunction, regulatory T cells

## Abstract

**Introduction:**

Childhood obesity may elevate the risk for adult obesity because of chronic inflammation and immune dysregulation; however, studies on the immune system in obese children are limited. This study aimed to investigate peripheral blood lymphocyte subpopulations in obese children and their association with metabolic markers.

**Methods:**

This prospective study included 30 obese children (median age: 11.5 years) and 22 age-matched non-obese controls. Lymphocyte populations were compared between the two groups. Additionally, factors influencing these populations were assessed in the obese group.

**Results:**

The proportion of CD4^+^ T cells and the ratio of naïve to memory CD4^+^ T cells were significantly lower in obese children than in non-obese children (*P* = 0.006 and *P* = 0.035, respectively). Regulatory T (Treg) cell counts were significantly higher in obese children than in non-obese children (*P* = 0.049). The proportion of CD4^+^ memory T cells positively correlated with serum alanine aminotransferase levels. The proportion of Tregs was negatively correlated with serum insulin levels and positively correlated with blood glucose levels. The number of CD4^+^ T cells and the ratio of naïve to memory CD4^+^ T cells were lower in obese children with a disease duration of <5 years compared to that in non-obese children. However, obese children with a disease duration of ≥5 years had a high number of CD4^+^ T cells.

**Conclusions:**

This study highlights the impact of childhood obesity on immune dysregulation. The changes in T cell subpopulations in obese children differ from those observed in obese adults and vary depend on the duration of obesity.

## 1 Introduction

Obesity is a major public health concern worldwide (1), with an increasing prevalence among adults and children. According to 2024 World Health Organization (WHO) data, the global obesity rate among children aged 5–19 years has quadrupled from 2% to 8% between 1990 and 2022. Lifestyle changes during the COVID-19 pandemic have further increased obesity rates (2). Childhood obesity has been associated with long-term health risks, including an increased likelihood of cardiovascular disease, diabetes, and other lifestyle-related diseases (3–7), as well as the morbidity and severity of infectious diseases (8, 9).

Obesity is also associated with immunological abnormalities. In obese adults, an increase in local effector and memory CD8^+^ T cells has been observed in adipose tissue, while a selective increase in CD4^+^ T cells, including naïve, memory, and regulatory T (Treg) cells, occurs in the peripheral blood (10, 11). This excessive T cells activity leads to the secretion of inflammatory cytokines, such as interleukin (IL)-2 and tumor necrosis factor (TNF)-α, triggering an inflammatory response within the tissues. This response has been linked to the development of insulin resistance and obesity-related diseases (12–15). Furthermore, excessive cytokine secretion can lead to immune system dysfunction, which predisposes patients to severe infections (15). While adult obesity has been linked to immune abnormalities, studies on the immune system in children remain limited.

In this study, we aimed to investigate the peripheral blood lymphocyte subpopulation in obese children and the association between each subpopulation and metabolic parameters.

## 2 Materials and methods

### 2.1 Participants

This prospective study included 30 obese children (18 males and 12 females; median age of 11.5 years; range, 6–16 years) who visited the Pediatric Endocrinology Outpatient Clinic of the Department of Pediatrics at the Hospital of the University of Occupational and Environmental Health, Japan, between July 2022 and December 2023. Obesity was defined as a body mass index (BMI)-standard deviation score (SDS) ≥2.0 according to the World Health Organization (WHO) criteria. BMI and BMI-SDS were calculated using national statistics for Japanese school children for 2000 (Ministry of Health, Labour and Welfare) (16). Twenty-two age-matched non-obese children (9 males and 13 females) who visited the same clinic during the study period but did not require treatment (e.g., idiopathic short stature) were included as controls. The height, weight, blood pressure, and laboratory data of all eligible children were extracted from electronic medical records. The primary objective of the study was to compare lymphocyte populations between the obese and control groups, while the secondary objective was to evaluate the factors influencing lymphocyte populations within the obese group.

### 2.2 Analyses of T and B cell subpopulations

Peripheral blood samples were collected from both obese children and controls. Subsequently, flow cytometry was performed on whole-blood specimens within 24 hours of collection, using ethylenediaminetetraacetic acid (EDTA) as an anticoagulant.

To determine the proportions of T and B cells in the gated lymphocytes, phycoerythrin (PE)-conjugated anti-CD3 antibody (Beckman Coulter, Brea, CA, USA) and fluorescein isothiocyanate (FITC)-conjugated anti-CD19 antibody (BD Biosciences, Mountain View, CA, USA) were used. For identifying helper and cytotoxic T cells, FITC-conjugated anti-CD4, PE-conjugated anti-CD3, and phycoerythrin-cyanin 5.1 (PC5)-conjugated anti-CD8 (Beckman Coulter) antibodies were applied. FITC-conjugated anti-CD45RA, PE-CD45RO PC5- CD4/CD8, and PC7-CD3 antibodies were used to define memory and naïve T cell subsets. B cell subsets were identified using FITC-conjugated IgD (BD Biosciences), PE-conjugated CD27, and PC5-conjugated CD19 (Beckman Coulter). Samples were incubated with fluorochrome-conjugated antibodies for 30 minutes in the dark and treated with a TQ-prep Workstation (Beckman Coulter) to lyse red cell before analysis.

Regulatory T cells (Tregs) were analyzed using the TregFlowEx Kit (EXBIO Praha, a.s., Vestec, Czech Republic). First, FITC-conjugated anti-CD4 and PE-conjugated anti-CD25 antibodies were added to blood samples and incubated for 10 minutes in the dark. Subsequently, the leukocytes were then permeabilized, stained intracellularly with an allophycocyanin-conjugated anti-FOXP3 antibody, and incubated for 30 minutes in the dark.

**Figure 1:**
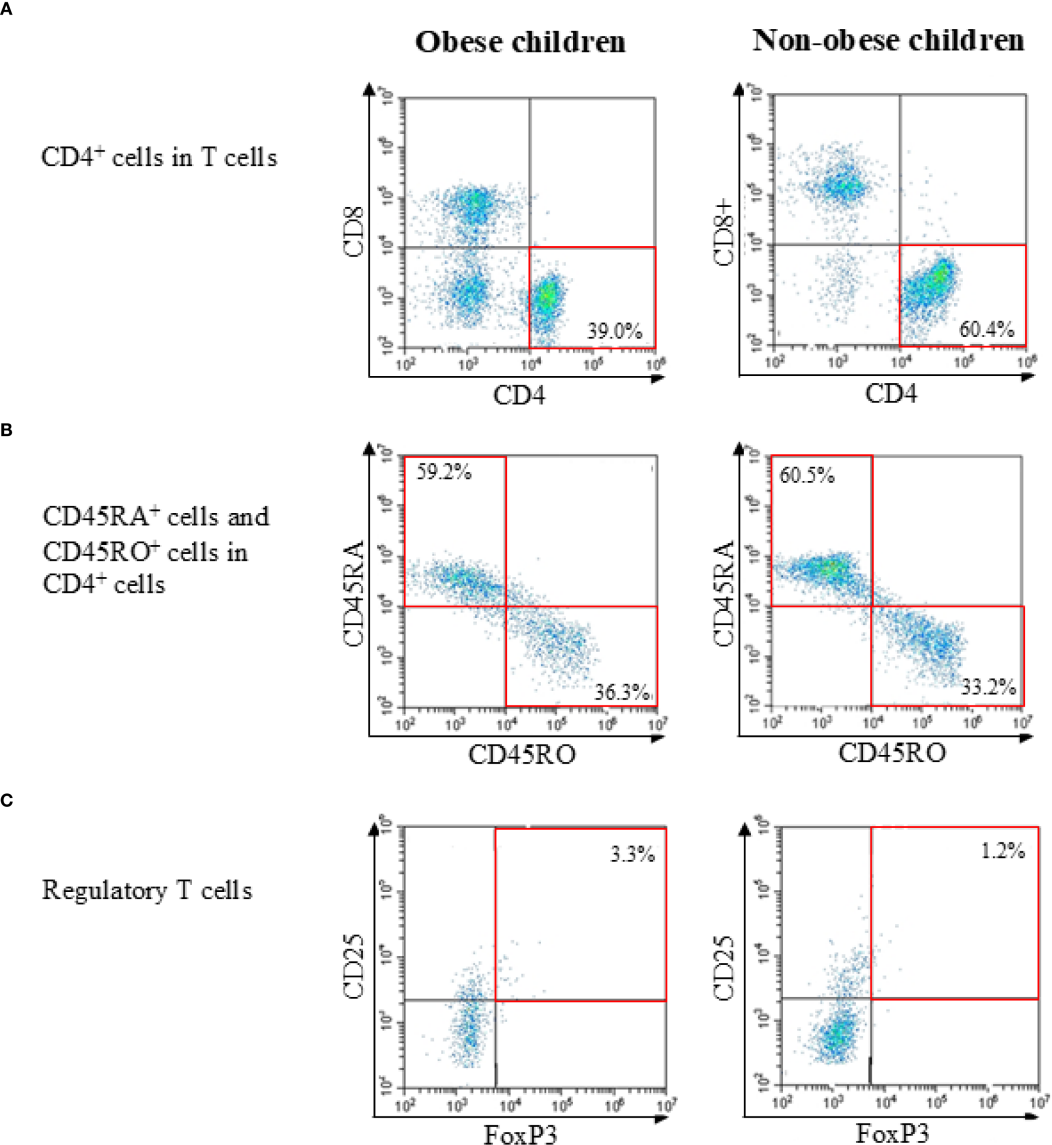
Representative flow cytometric gating strategies in obese child group and non-obese child group. **(A)** Identification of T cells within lymphocytes and separation into CD4^+^ and CD8^+^ subsets. **(B)** Analysis of naïve (CD45RA^+^) and memory (CD45RO^+^) subsets within CD4^+^ T cells. **(C)** Identification of regulatory T cells (CD4^+^CD25^+^FOXP3^+^) within CD4^+^ T cells. Percentages and representative values are shown in each plot.

Two-, three-, or four-color flow cytometry was performed using a CytoFLEX cytometer (Beckman Coulter). The specificity of staining was confirmed using fluorochrome-conjugated isotype-matched monoclonal antibodies. To ensure accurate discrimination of cell populations, the fluorescence minus one control was used to set gating boundaries, and single-stained compensation controls were applied to correct for spectral overlap between channels. Instrument settings were standardized and calibrated daily using manufacturer-provided fluorescent calibration beads. Lymphocytes were first gated based on forward and side scatter profiles, followed by identification of CD3^+^ T cells, which were then separated into CD4^+^ and CD8^+^ subsets. Subsequent gating distinguished naïve, memory, and regulatory T cell populations. All analyses, except for Tregs, were performed using at least 20,000 cells. For Treg analysis, at least 40,000 cells were used. A representative flow cytometric gating strategy for identifying lymphocyte subsets, including CD3^+^ T cells, CD4^+^ and CD8^+^ subsets, naïve (CD45RA^+^) and memory (CD45RO^+^) CD4^+^ T cells, and regulatory T cells (CD4^+^CD25^+^FOXP3^+^), is shown in **Figure 1**.

### 2.3 Statistical analysis

SPSS statistical software ver. 23.0 (IBM Corp., Armonk, NY, USA) was used for statistical analyses. The Mann–Whitney U test was used to compare the differences between the quantitative values, and Fisher’s exact test was applied to compare the qualitative data. Spearman’s correlation coefficient and multiple regression analysis were used to examine factors associated with lymphocyte subsets in the obese group. *P*-values <0.05 were considered statistically significant.

### 2.4 Ethical approval

The Institutional Review Board of the University of Occupational and Environmental Health, Japan (UOEHCRB21-073) approved this study. Informed consent was obtained from the parents of all patients.

## 3 Results

### 3.1 Background of eligible children

The demographic and clinical characteristics of the eligible children are summarized in **Table 1**. The median disease duration among obese children was 42 months (a quartile range of 27–68 months). Weight, BMI, BMI-SDS, and blood pressure were significantly higher in the obese children than in the non-obese children. Among laboratory markers, peripheral white blood cell (WBC) counts and serum levels of aspartate aminotransferase, alanine aminotransferase (ALT), triglycerides, total cholesterol, and insulin were significantly higher in obese children than in non-obese children. The median (interquartile range) HOMA-IR was 3.4 (2.3–8.1) in obese children and 1.6 (1.3–2.0) in non-obese children (<0.001), indicating increased insulin resistance in the obese group. HOMA-β was also calculated for reference, showing no evidence of impaired insulin secretion; however, as insulin resistance was the primary clinical parameter of interest, only HOMA-IR values are presented in **Table 1**.

**Table 1:** Comparisons of demographic and clinical characteristics and laboratory findings between obese and non-obese children.

	Obese children (n = 30)	Non-obese children (n = 22)	*P*-value
Age, years*^1^	11.5 (10.0-13.8)	10.6 (9.1-15.1)	0.337
Gender, boys/girls	18/12	9/13	0.173
Height, cm*^1^	154.0 (140.0-164.2)	142.3 (132.8-159.5)	0.071
Height-standard deviation score*^1^	0.7 (0.5-1.3)	1 (-0.7-0.6)	<0.001
Weight, kg*^1^	69.9 (50.0-88.5)	40.2 (31.5-50.9)	<0.001
Body mass index*^1^	29.8 (25.7-32.6)	19.6 (17.0-20.6)	<0.001
Body mass index-standard deviation score*^1^	2.6 (2.0-2.7)	0.5 (-0.4-0.9)	<0.001
Systolic blood pressure, mmHg*^1^	120 (114-128)	102 (95-117)	<0.001
Diastolic blood pressure, mmHg*^1^	73 (67-77)	67 (57-70.5)	0.003
Disease duration, months*^1^	42 (27-68)	N/A	–
Peripheral white blood cell counts,/µL*^1^	7359 (6700-9175)	5900 (4925-7150)	<0.001
Serum aspartate aminotransferase levels, U/L*^1^	25.5 (20.3-39.5)	21.5 (17.3-25.8)	0.014
Serum alanine aminotransferase levels, U/L*^1^	42.5 (24.3-74.3)	15 (12.0-18.5)	<0.001
Serum triglycerides levels, mg/dL*^1^	129 (88.0-168.5)	70 (53.8-90.5)	<0.001
Serum total cholesterol levels, mg/dL*^1^	178 (157-200)	163 (149-184)	0.108
Blood glucose levels, mg/dL*^1^	95 (90.8-102.8)	98 (91.5-102.5)	0.406
Hemoglobin A1c test, %*^1^	5.7 (5.5-5.8)	5.5 (5.5-5.7)	0.489
Serum insulin levels, µU/mL*^1^	15.1 (10.7-31.2)	7.1 (5.3-7.8)	<0.001
HOMA-IR*^1^	3.4 (2.3-8.1)	1.6 (1.3-2.0)	<0.001

*^1^Data is represented as the median (interquartile range).

N/A, not applicable; HOMA-IR, homeostasis model assessment of insulin resistance.

### 3.2 Lymphocyte subpopulations in obese children

To analyze the characteristics of lymphocyte subpopulations in obese children, we compared lymphocyte subpopulations between obese and non-obese children using flow cytometry. Peripheral WBC counts, lymphocyte counts, and the proportions and absolute numbers of T and B cell subpopulations in obese and non-obese children are presented in **Table 2**. Peripheral WBC counts were significantly higher in obese children than in non-obese children, whereas lymphocyte counts did not differ significantly between the two groups. The proportions of T cells (among lymphocytes) and CD4^+^ T cells were significantly lower in obese children than in non-obese children (T cells: 52.3% vs. 60.7%, *P* = 0.011; CD4^+^ T cells: 39.0% vs. 50.4%, *P* = 0.006). The absolute numbers of T cells and CD4^+^ T cells were also lower in obese children, but this difference was not statistically significant. Among CD4^+^ T cells, the ratio of CD45RA^+^ cells (naïve CD4^+^ T cells) to CD45RO^+^ cells (memory CD4^+^ T cells) was significantly lower in obese children than in non-obese children (1.7 vs. 1.9, *P* = 0.035). The proportion and the absolute number of Tregs in obese children (proportion, 3.9%; counts, 20 cells/µL) were significantly higher than those in non-obese children (proportion, 1.9%; counts, 11 cells/µL) (proportion, *P* < 0.001; counts, *P* = 0.049). There were no significant differences in the proportion or absolute numbers of CD8^+^ T or B cells between the two groups. Furthermore, there was no significant difference observed in the proportion of B cell subpopulations between the two groups.

**Table 2:** Comparison of lymphocyte subpopulations between obese and non-obese children.

	Obese children (n = 30)	Non-obese children (n = 22)	*P*-value
Peripheral white blood cell counts, cells/µL*^1^	7359 (6700-9175)	5900 (4925-7150)	<0.001
Lymphocyte counts, cells/µL*^1^	2470 (2173-3018)	2402 (1926-3074)	0.390
T cells*^2^			
Counts, cells/µL*^1^	1255 (1146-1387)	1334 (1064-1897)	0.210
Percentage in lymphocytes, %*^1^	52.3 (47.7-57.1)	60.7 (52.4-63.2)	0.011
CD4^+^ cells			
Counts, cells/µL*^1^	448 (337-715)	648 (482-773)	0.130
Percentage in T cells, %*^1^	39.0 (32.4-51.5)	50.4 (45.6-56.5)	0.006
CD45RA^+^ cells/CD45RO^+^ cells in CD4^+^ cells*^1^	1.7 (1.1-2.0)	1.9 (1.3-2.7)	0.035
CD8^+^ cells			
Counts, cells/µL*^1^	424 (287-536)	392 (304-536)	0.320
Percentage in T cells, %*^1^	31.4 (27.3-38.9)	32.0 (29.8-34.8)	0.376
CD45RA^+^ cells/CD45RO^+^ cells in CD8^+^ cells*^1^	3.0 (2.1-4.3)	3.6 (2.4-4.9)	0.111
Regulatory T cells			
Counts, cells/µL*^1^	20 (12-33)	11 (3-17)	0.049
Percentage in CD4^+^ cells, %*^1^	3.9 (2.6-5.9)	1.9 (0.5-3.1)	<0.001
B cells*^3^			
Counts, cells/µL*^1^	398 (293-528)	377 (238-445)	0.173
Percentage in lymphocytes, %*^1^	15.9 (13.1-17.7)	14.4 (11.9-17.3)	0.143
Percentage of IgD^+^CD27^-^ cells in B cells, %*^1^	68.5 (48.7-75.8)	70.8 (66.6-73.1)	0.053
Percentage of IgD^+^CD27^+^ cells in B cells, %*^1^	3.8 (2.1-7.0)	6.1 (3.3-8.7)	0.053
Percentage of IgD^-^CD27^+^ cells in B cells, %*^1^	11.3 (7.3-13.5)	11.8 (9.4-13.9)	0.170

*^1^Data is represented as the median (interquartile range).

*^2^T cells were identified by flow cytometry, using phycoerythrin-conjugated anti-CD3 antibody.

*^3^B cells were identified by flow cytometry, using fluorescein isothiocyanate-conjugated anti-CD19 antibody.

### 3.3 Correlations between CD4^+^ T cell subpopulations and metabolic parameters in obese children

**Figure 2:**
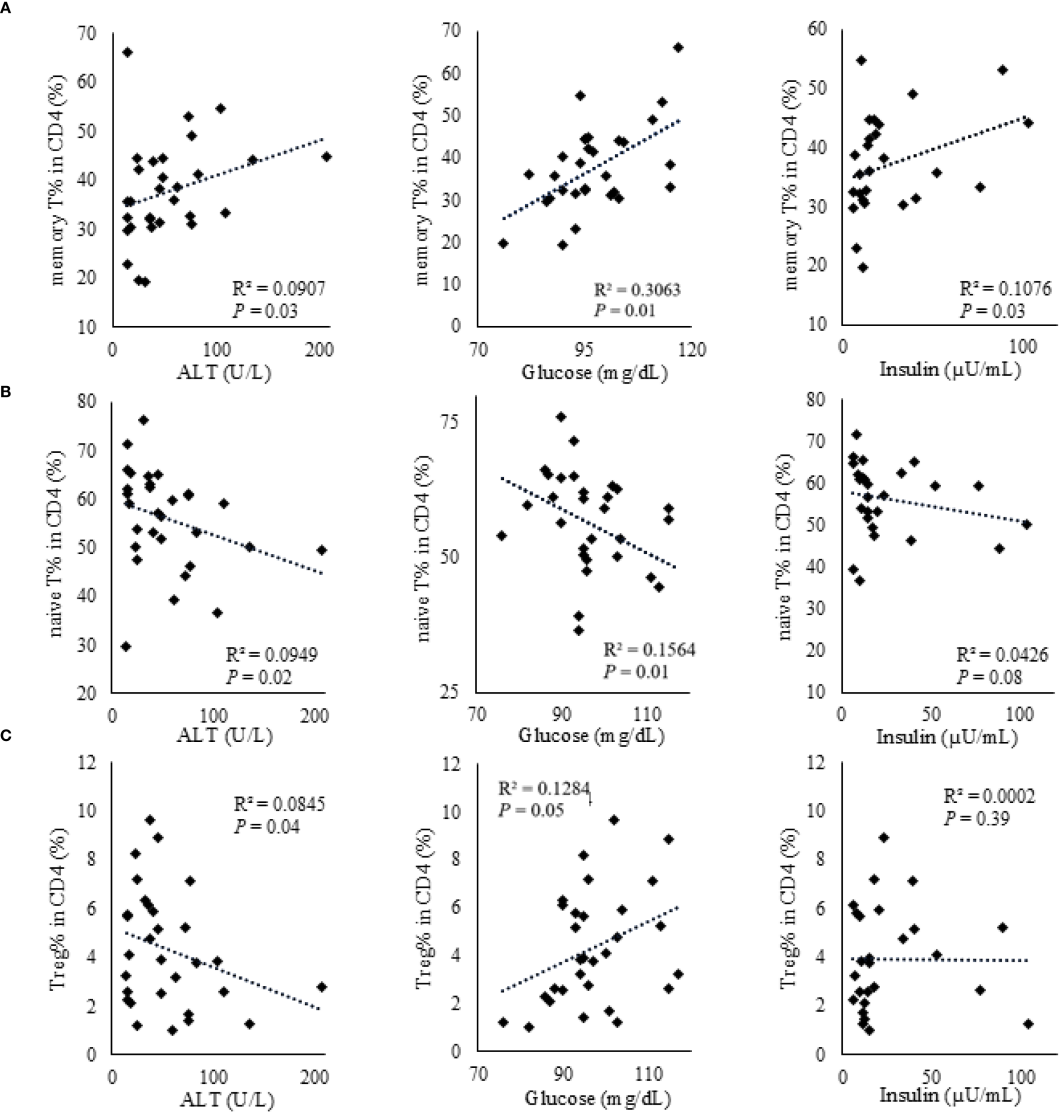
Correlations between CD4^+^ T cell subsets and metabolic parameters (ALT, glucose, and insulin) in obese children. **(A)** Memory CD4^+^ T cells. **(B)** Naïve CD4^+^ T cells. **(C)** Regulatory T cells. Correlations with serum ALT (U/L), glucose (mg/dL), and insulin (µU/mL) are shown in each panel. Associations were assessed using Spearman’s rank correlation; ρ and *P*-values are displayed. Each dot represents an individual participant, and fitted lines are shown for visual guidance.

We investigated the correlation between CD4^+^ T cell subpopulations and anthropometric and metabolic parameters in obese children. **Figure 2** was reorganized by cell subsets to clarify these associations. The proportion of CD4^+^ memory T cells showed a significant positive correlation with serum ALT and blood glucose levels but a significant negative correlation with serum insulin levels (**Table 3**, **Figure 2A**). The proportion of naïve CD4^+^ T cells exhibited a significant negative correlation with serum ALT and blood glucose levels and a significant positive correlation with serum insulin levels (**Table 3**, **Figure 2B**). Furthermore, the proportion of Tregs showed a significant negative correlation with serum ALT levels (**Table 3**, **Figure 2C**).

**Table 3:** Correlation between CD4^+^ T cell subpopulations and anthropometric or metabolic parameters in obese children.

Parameter	CD4^+^ memory T cells*^1,2^		CD4^+^ naive T cells*^1,3^		Regulatory T cells*^1,4^	
	R	*P*-value	R	*P*-value	R	*P*-value
Age	0.18	0.35	-0.11	0.58	-0.13	0.51
Body mass index	0.22	0.23	-0.16	0.40	0.13	0.51
Body mass index-standard deviation score	0.03	0.87	-0.12	0.52	0.22	0.24
Systolic blood pressure	0.04	0.83	0.05	0.81	0.18	0.34
Diastolic blood pressure	-0.20	0.28	0.31	0.09	-0.05	0.79
Serum aspartate aminotransferase levels	0.29	0.12	-0.32	0.09	-0.23	0.22
Serum alanine aminotransferase levels	0.41	0.03	-0.42	0.02	-0.39	0.04
Serum triglycerides levels	0.07	0.73	-0.08	0.68	0.18	0.35
Serum total cholesterol levels	-0.21	0.27	0.09	0.63	-0.16	0.41
Blood glucose levels	0.49	0.01	-0.45	0.01	0.36	0.05
Hemoglobin A1c test	0.13	0.52	0.00	0.99	0.34	0.07
Serum insulin levels	0.43	0.03	-0.35	0.08	0.18	0.39

Median values (interquartile ranges) for clinical parameters (e.g., age, body mass index, blood pressure, biochemical variables) are presented in **Table 1**, and those for lymphocyte subsets, including CD4^+^ memory T cells, CD4^+^ naive T cells, and regulatory T cells, are presented in **Table 2**.

*^1^These represent the percentage of each cell in the CD4^+^ T cells.

*^2^CD4^+^ memory T cells were identified by flow cytometry, using fluorescein isothiocyanate-conjugated anti-CD45RA, phycoerythrin-cyanin 5.1-conjugated anti-CD4 and phycoerythrin-cyanin 7-conjugated anti-CD3 antibodies.

*^3^CD4^+^ naive T cells were identified by flow cytometry, using phycoerythrin-conjugated anti-CD45RO, phycoerythrin-cyanin 5.1-conjugated anti-CD4 and phycoerythrin-cyanin 7-conjugated anti-CD3 antibodies.

*^4^Regulatory T cells were identified by flow cytometry, using fluorescein isothiocyanate-conjugated anti-CD4 and phycoerythrin-conjugated anti-CD25, and allophycocyanin-conjugated anti-FOXP3 antibodies.

In the multivariate analysis, the proportion of memory CD4^+^ T cells showed a significant positive correlation with serum ALT levels (**Table 4**), while the proportion of naïve CD4^+^ T cells was significantly negatively correlated with serum ALT levels. Additionally, Treg counts showed a significant negative correlation with serum levels of ALT and insulin and a significant positive correlation with blood glucose levels.

**Table 4:** Multivariate analysis of the correlation between CD4^+^ T cell subpopulations and metabolic parameters in obese children.

Parameter	β	95% confidence interval	*P*-value	R^2^
CD4^+^ memory T cell*^1,2^				0.69
Serum alanine aminotransferase levels	0.40	0.002 - 0.148	0.044	
Blood glucose levels	0.36	-0.081 - 0.709	0.113	
Serum insulin levels	-0.04	-0.161 - 0.135	0.855	
CD4^+^ naive T cell*^1,3^				0.56
Serum alanine aminotransferase levels	-0.45	-0.167 - -0.006	0.037	
Blood glucose levels	-0.14	-0.559 - 0.313	0.565	
Serum insulin levels	0.04	-0.149 - 0.178	0.857	
Regulatory T cell*^1,4^				0.75
Serum alanine aminotransferase levels	-0.40	-0.035 - -0.004	0.017	
Blood glucose levels	0.90	0.117- 0.285	<0.001	
Serum insulin levels	-0.41	-0.066 - -0.003	0.034	

Median values (interquartile ranges) for laboratory data are presented in **Table 1**, and those for lymphocyte subsets, including CD4^+^ memory T cells, CD4^+^ naive T cells, and regulatory T cells, are presented in **Table 2**.

*^1^These represent the percentage of each cell in the CD4^+^ T cells.

*^2^CD4^+^ memory T cells were identified by flow cytometry, using fluorescein isothiocyanate-conjugated anti-CD45RA, phycoerythrin-cyanin 5.1-conjugated anti-CD4 and phycoerythrin-cyanin 7-conjugated anti-CD3 antibodies.

*^3^CD4^+^ naive T cells were identified by flow cytometry, using phycoerythrin-conjugated anti-CD45RO, phycoerythrin-cyanin 5.1-conjugated anti-CD4 and phycoerythrin-cyanin 7-conjugated anti-CD3 antibodies.

*^4^Regulatory T cells were identified by flow cytometry, using fluorescein isothiocyanate-conjugated anti-CD4 and phycoerythrin-conjugated anti-CD25, and allophycocyanin-conjugated anti-FOXP3 antibodies.

### 3.4 Changes in lymphocyte subpopulations with duration of obesity

We classified obese children into two groups: those with a disease duration of ≥5 years (n = 9) and those with a disease duration of <5 years (n = 21) and compared their lymphocyte subpopulations. Peripheral lymphocyte counts and the absolute numbers of T cells were significantly higher in obese children with a disease duration of ≥5 years compared to that in those with a disease duration of <5 years (lymphocytes: 2,917 vs. 2,297/µL, *P* = 0.026; T cells: 1,390 vs. 1,213/µL, *P* = 0.033) (**Table 5**). The absolute numbers of CD4^+^ cells and Tregs and the proportion of CD4^+^ T cells were significantly higher in obese children with a disease duration of ≥5 years (CD4^+^ cells: 776 vs. 415/µL, *P* = 0.022; Tregs: 35 vs. 17/µL, *P* = 0.024; the proportion of CD4^+^ T cells: 51.9% vs. 34.3%, *P* = 0.018). The proportion of Tregs in CD4^+^ T cells was higher in obese children with a disease duration of ≥5 years (5.2%) than in those with a disease duration of <5 years (3.3%); however, the difference was not statistically significant. The absolute numbers of CD8^+^ T or B cells did not differ between the two groups (data not shown).

**Table 5:** Comparison of lymphocyte subpopulations in obese children between obesity duration < 5 years and ≥5 years.

	Obesity duration <5 years (n = 21)	Obesity duration ≥ 5years (n = 9)	*P*-value
Peripheral white blood cell counts, cells/µL*^1^	7200 (6700-8500)	8200 (7400-9800)	0.198
Lymphocyte counts, cells/µL*^1^	2297 (2093-2633)	2917 (2545-3538)	0.026
T cells*^2^			
Counts, cells/µL*^1^	1213 (1044-1325)	1390 (1318-1924)	0.033
Percentage in lymphocytes, %*^1^	52.3 (47.3-56.5)	52.4 (49.6-57.2)	0.264
CD4^+^ cells			
Counts, cells/µL*^1^	415 (332-575)	776 (493-942)	0.022
Percentage in T cells, %*^1^	34.3 (30.2-44.1)	51.9 (40.1-61.6)	0.008
CD45RA^+^ cells/CD45RO^+^ cells in CD4^+^ cells*^1^	1.7 (1.1-2.0)	1.8 (1.2-2.1)	0.184
Regulatory T cells			
Counts, cells/µL*^1^	17 (11-27)	35 (15-66)	0.024
Percentage in CD4^+^ cells, %*^1^	3.3 (2.3-5.7)	5.2 (3.9-6.3)	0.098

*^1^Data is represented as the median (interquartile range).

*^2^T cells were identified by flow cytometry, using phycoerythrin-conjugated anti-CD3 antibody.

**Figure 3:**
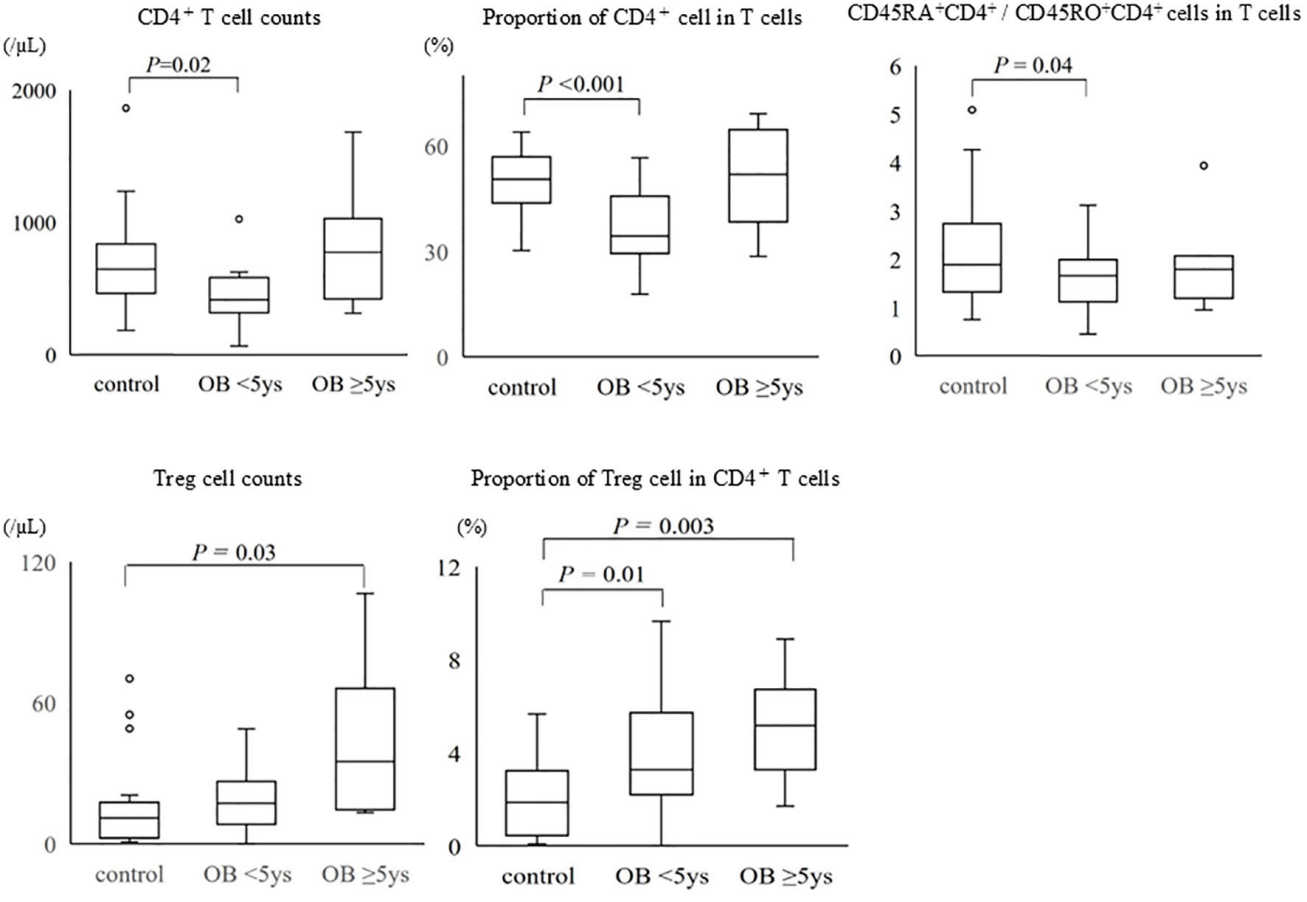
Comparison of CD4^+^ T cells and regulatory T cells among the groups of non-obese children, obese children with disease duration of <5 years, and those with disease duration of ≥5 years. The bottom and the top of the box correspond to the 25th and 75th percentile points, respectively. The line within the box represents the median, the whiskers indicate the values of the 10th and 90th percentiles, and the open circles represent the outlier values beyond the 10th and 90th percentiles. Control: non-obese children, OB <5 years: obese children with disease duration of <5 years, OB ≥5 years: obese children with disease duration of ≥5 years, Treg: regulatory T cell.

The absolute number of CD4^+^ T cells, the proportion of CD4^+^ T cells, and the ratio of naïve CD4^+^ T cells to memory CD4^+^ T cells were lower in obese children with disease duration of <5 years than in non-obese children, whereas obese children with disease duration of ≥5 years had a higher absolute number of CD4^+^ T cells and proportion of CD4^+^ T cells than did non-obese children (**Figure 3**). The absolute number of Tregs and the proportion of Tregs in CD4^+^ T cells were higher in obese children than in non-obese children, regardless of disease duration, and these differences were particularly pronounced in obese children with a disease duration of ≥5 years.

## 4 Discussion

This study demonstrated that T cell subpopulations in the peripheral blood differed between obese and non-obese children. Specifically, the proportion of CD4^+^ T cells and the ratio of naïve CD4^+^ T cells to memory CD4^+^ T cells were significantly lower in obese children; however, the obese children had significantly higher Treg cell counts. Additionally, in obese children, serum ALT levels were strongly associated with changes in memory CD4^+^ T cells, naïve CD4^+^ T cells, and Tregs, and there was a positive correlation between the proportion of Tregs and serum glucose levels. These changes in T cell subpopulations in obese children are different from those seen in obese adults (10) and may vary depending on the duration of obesity. This study suggests that immune dysregulation in childhood obesity is progressive and that early intervention can help improve it.

Previous studies have shown that the proportion of Tregs is reduced in obese adults and patients with impaired glucose tolerance (17, 18). In adult obesity, Tregs in the adipose tissue are also reduced (19), leading to an inadequate ability to suppress inflammation. However, in this study, the number of Tregs in the peripheral blood was found to be increased in obese children. This variation maybe a specific feature of childhood obesity. Childhood obesity typically involves milder inflammation than that in adult obesity (20), and an increase in Tregs may represent a response to maintain inflammatory homeostasis. Similar to the phenomenon in obese children, Tregs in the peripheral blood and intestinal tissues are increased in children with inflammatory bowel disease (21). Considering these results, the number of Tregs may increase in children as a compensatory measure to suppress chronic inflammation, suggesting that Treg cell dynamics and functional abnormalities differ between children and adults.

Analysis of the relationship between the proportion of Tregs and blood glucose and serum insulin levels revealed a positive correlation with the former and a negative correlation with the latter. In childhood obesity, mild elevations in blood glucose levels may reflect an early phase of low-grade chronic inflammation, which may induce compensatory Treg cell proliferation. At this stage, immune regulatory mechanisms are relatively intact, suggesting active feedback suppression of emerging inflammation. In contrast, elevated serum insulin levels possibly indicate a more advanced stage of insulin resistance and metabolic dysfunction, where persistent chronic inflammation may induce Treg dysfunction or depletion. In adult obesity, persistent hyperglycemia and chronic inflammation have been associated with a reduction in Treg counts, contributing to the breakdown of immune regulatory networks and the establishment of meta-inflammation (17–19). The loss of Tregs in the visceral adipose tissue has been implicated in the progression of insulin resistance and metabolic syndrome (17). Although Treg inducibility is preserved during the early stages of childhood obesity, sustained elevation of blood glucose levels may impair this compensatory mechanism, leading to Treg dysfunction and depletion. In this study, the longer the duration of obesity, the more significant the increase in Treg counts. Long-term observations are required to validate our hypothesis regarding the effects of blood glucose and insulin levels on Tregs.

In this study, the proportion of CD4^+^ T cells and the ratio of naïve CD4^+^ T cells to memory CD4^+^ T cells were significantly lower in obese children than in non-obese children, whereas the proportion of CD4^+^ T cells was significantly higher in obese children with disease duration of ≥5 years than in those with disease duration of <5 years. In addition, when limited to obese children with a disease duration of ≥5 years, the absolute CD4^+^ T cell counts exceeded those observed in non-obese children. In adult obesity, chronic low-grade inflammation drives the persistent activation of naïve CD4^+^ T cells, leading to their exhaustion and depletion, followed by the expansion of memory CD4^+^ T cells. This phenomenon is closely associated with adipose tissue inflammation, systemic metabolic dysfunction, and the development of insulin resistance (12). In contrast, the immunological trajectory of pediatric obesity remains poorly characterized. In children, the immune system is developing, with a predominance of naïve T cells and a relatively limited memory T cell pool (22). Moreover, mechanisms promoting immune tolerance to self and environmental antigens are highly active during childhood, potentially limiting the degree of memory T cell expansion in response to chronic stimuli (22). Therefore, it is unclear whether chronic inflammation in pediatric obesity induces immune remodeling similar to that observed in adults. Our study provides novel evidence that in obese children, non-Treg CD4^+^ T cells are initially reduced–possibly reflecting early immune exhaustion, but gradually recover over time through the accumulation of memory CD4^+^ T cells. These findings suggest pediatric obesity is a distinct, dynamic, and developmentally regulated phase of immune remodeling that differs from the immunopathology seen in adult obesity.

A notable finding of this study was that serum ALT levels were positively and negatively correlated with the proportions of memory and naïve CD4^+^ T cells, respectively. Obesity-related fatty liver disease (non-alcoholic fatty liver disease) and hepatocyte injury increase the production of inflammatory cytokines such as interleukin-6 (IL-6) and tumor necrosis factor-alpha (TNF-α) (23, 24). These inflammatory cytokines may chronically stimulate T cells and promote the differentiation of naïve CD4^+^ T cells, causing an increase in the number of memory CD4^+^ T cells. Furthermore, the effect of aberrant liver function on T-cell differentiation has been reported in adults, with elevated ALT levels reflecting chronic inflammation and altered immune responses (25). These results suggest that liver dysfunction is a crucial factor in the progression of immune dysregulation during childhood obesity.

In this study, no significant differences were observed between B and CD8^+^ T cells between obese and non-obese children. This suggests that these cell types may be less susceptible to immune changes during the early stages of inflammation in childhood obesity. Although the activation of CD8^+^ T cells exacerbates adipose tissue inflammation in adult obesity (26, 27), immunological changes in the adipose tissue are less advanced in children than in adults, and their effects on CD8^+^ T and B cells possibly remain less significant. Further studies are required to determine the maturity of the immune system and the influence of environmental factors on childhood.

This study had some limitations. First, this was a single-center study with a small sample size. Although future multicenter studies with larger populations are necessary to improve the reliability and generalizability of the results, *post-hoc* power analyses for major outcome variables (e.g., CD4^+^ memory T cell proportion, regulatory T cell proportion, and serum insulin) indicated sufficient statistical power (≥0.95) to detect significant differences. This suggests that the present dataset is adequate to support the study conclusions despite the limited sample size. Second, the analysis of lymphocyte subsets was based on peripheral blood and did not directly involve immune cell infiltration or dynamics in adipose tissue. In adult obesity, immune cells in the adipose tissue play a crucial role in chronic inflammation; however, it is unknown if a similar mechanism exists in children. Finally, inflammatory cytokines were not measured in the present study. IL-6 and TNF-α promote chronic low-grade inflammation and impair insulin signaling, which can increase susceptibility to metabolic problems from childhood (28–30). Previous pediatric studies have already reported elevated levels of proinflammatory cytokines such as IL-6 and TNF-α, but the inclusion of cytokine measurements to this study could have allowed a more comprehensive evaluation of immune function. Future studies should consider combining immune cell phenotyping with cytokine profiling to better characterize immunometabolic interactions in pediatric obesity.

This study showed that childhood obesity is associated with immune dysregulation, characterized by a decreased ratio of naïve CD4^+^ T cells to memory CD4^+^ T cells and increased Treg counts. These changes were strongly correlated with liver dysfunction (ALT elevation). Furthermore, the duration of obesity may influence changes in T cell subpopulations. Chronic inflammation may drive these immune abnormalities, potentially increasing the susceptibility to infections. Early intervention is crucial to preventing long-term immune dysregulation and the associated health risks. Further studies are needed to clarify mechanisms underlying immune changes and their impact on pediatric health.

In conclusion, these findings provide further evidence that immunological changes in childhood obesity are not static but evolve with disease duration. While our study evaluated immune cells only in peripheral blood, the association between T cell shifts and serum ALT levels implies that hepatic inflammation may play a key role in initiating or perpetuating immune remodeling. These findings suggest a potential window for immune intervention before irreversible immunometabolic alterations occur.

## Data Availability

The datasets presented in this article are not readily available because the datasets generated for this study are not available due to ethical restrictions and institutional policy prohibiting the sharing of individual-level clinical data, even in anonymized form. Requests to access the datasets should be directed to Reiko Saito, reiko-z@med.uoeh-u.ac.jp.

## References

[1] (NCD-RisC). NRFC. Worldwide trends in body-mass index, underweight, overweight, and obesity from 1975 to 2016: a pooled analysis of 2416 population-based measurement studies in 128·9 million children, adolescents, and adults. Lancet. (2017) 390:2627–42. doi: 10.1016/s0140-6736(17)32129-3, PMID: 29029897 PMC5735219

[2] Suárez-Reyes M Fernández-Verdejo R Quintiliano D Pinheiro AC Pizarro T. Effects of school closure on lifestyle behaviours and health outcomes in children during the COVID-19 pandemic in Chile: A time-matched analysis. Pediatr Obes. (2024) 19:e13182. doi: 10.1111/ijpo.13182, PMID: 39379176 PMC11560478

[3] Badeli H Hassankhani A Naeemi Z Hosseinzadeh S Mehrabi S Pourkarimi M. Prevalence of hypertension and obesity-related hypertension in urban school-aged children in rasht. Iran J Kidney Dis. (2016) 10:364–8., PMID: 27903995

[4] Boyd GS Koenigsberg J Falkner B Gidding S Hassink S. Effect of obesity and high blood pressure on plasma lipid levels in children and adolescents. Pediatrics. (2005) 116:442–6. doi: 10.1542/peds.2004-1877, PMID: 16061601

[5] Dieli F Poccia F Lipp M Sireci G Caccamo N Di Sano C. Differentiation of effector/memory Vdelta2 T cells and migratory routes in lymph nodes or inflammatory sites. J Exp Med. (2003) 198:391–7. doi: 10.1084/jem.20030235, PMID: 12900516 PMC2194087

[6] Thompson DR Obarzanek E Franko DL Barton BA Morrison J Biro FM. Childhood overweight and cardiovascular disease risk factors: the National Heart, Lung, and Blood Institute Growth and Health Study. J Pediatr. (2007) 150:18–25. doi: 10.1016/j.jpeds.2006.09.039, PMID: 17188606 PMC1945042

[7] Tung JYL Poon GWK Du J Wong KKY. Obesity in children and adolescents: Overview of the diagnosis and management. Chronic Dis Transl Med. (2023) 9:122–33. doi: 10.1002/cdt3.58, PMID: 37305109 PMC10249183

[8] de Frel DL Atsma DE Pijl H Seidell JC Leenen PJM Dik WA. The impact of obesity and lifestyle on the immune system and susceptibility to infections such as COVID-19. Front Nutr. (2020) 7:597600. doi: 10.3389/fnut.2020.597600, PMID: 33330597 PMC7711810

[9] Maccioni L Weber S Elgizouli M Stoehlker AS Geist I Peter HH. Obesity and risk of respiratory tract infections: results of an infection-diary based cohort study. BMC Public Health. (2018) 18:271. doi: 10.1186/s12889-018-5172-8, PMID: 29458350 PMC5819164

[10] van der Weerd K Dik WA Schrijver B Schweitzer DH Langerak AW Drexhage HA. Morbidly obese human subjects have increased peripheral blood CD4+ T cells with skewing toward a Treg- and Th2-dominated phenotype. Diabetes. (2012) 61:401–8. doi: 10.2337/db11-1065, PMID: 22228716 PMC3266399

[11] Yuan Y Li H Liao Y Feng C. CD8+ T cells are involved in early inflammation before macrophages in a rat adipose tissue engineering chamber model. J Tissue Eng Regener Med. (2019) 13:1499–506. doi: 10.1002/term.2836, PMID: 30811089

[12] Chehimi M Vidal H Eljaafari A. Pathogenic role of IL-17-producing immune cells in obesity, and related inflammatory diseases. J Clin Med. (2017) 6:68. doi: 10.3390/jcm6070068, PMID: 28708082 PMC5532576

[13] McLaughlin T Ackerman SE Shen L Engleman E. Role of innate and adaptive immunity in obesity-associated metabolic disease. J Clin Invest. (2017) 127:5–13. doi: 10.1172/jci88876, PMID: 28045397 PMC5199693

[14] Weisberg SP McCann D Desai M Rosenbaum M Leibel RL Ferrante AW Jr. Obesity is associated with macrophage accumulation in adipose tissue. J Clin Invest. (2003) 112:1796–808. doi: 10.1172/jci19246, PMID: 14679176 PMC296995

[15] Wellen KE Hotamisligil GS. Inflammation, stress, and diabetes. J Clin Invest. (2005) 115:1111–9. doi: 10.1172/jci25102, PMID: 15864338 PMC1087185

[16] Isojima T Kato N Ito Y Kanzaki S Murata M. Growth standard charts for Japanese children with mean and standard deviation (SD) values based on the year 2000 national survey. Clin Pediatr Endocrinol. (2016) 25:71–6. doi: 10.1297/cpe.25.71, PMID: 27212799 PMC4860518

[17] Feuerer M Herrero L Cipolletta D Naaz A Wong J Nayer A. Lean, but not obese, fat is enriched for a unique population of regulatory T cells that affect metabolic parameters. Nat Med. (2009) 15:930–9. doi: 10.1038/nm.2002, PMID: 19633656 PMC3115752

[18] Winer S Chan Y Paltser G Truong D Tsui H Bahrami J. Normalization of obesity-associated insulin resistance through immunotherapy. Nat Med. (2009) 15:921–9. doi: 10.1038/nm.2001, PMID: 19633657 PMC3063199

[19] Bradley D Smith AJ Blaszczak A Shantaram D Bergin SM Jalilvand A. Interferon gamma mediates the reduction of adipose tissue regulatory T cells in human obesity. Nat Commun. (2022) 13:5606. doi: 10.1038/s41467-022-33067-5, PMID: 36153324 PMC9509397

[20] Carolan E Hogan AE Corrigan M Gaotswe G O’Connell J Foley N. The impact of childhood obesity on inflammation, innate immune cell frequency, and metabolic microRNA expression. J Clin Endocrinol Metab. (2014) 99:E474–8. doi: 10.1210/jc.2013-3529, PMID: 24423308

[21] Sznurkowska K Luty J Bryl E Witkowski JM Hermann-Okoniewska B Landowski P. Enhancement of circulating and intestinal T regulatory cells and their expression of helios and neuropilin-1 in children with inflammatory bowel disease. J Inflammation Res. (2020) 13:995–1005. doi: 10.2147/jir.S268484, PMID: 33273840 PMC7705274

[22] Simon AK Hollander GA McMichael A. Evolution of the immune system in humans from infancy to old age. Proc Biol Sci. (2015) 282:20143085. doi: 10.1098/rspb.2014.3085, PMID: 26702035 PMC4707740

[23] Tilg H Moschen AR. Inflammatory mechanisms in the regulation of insulin resistance. Mol Med. (2008) 14:222–31. doi: 10.2119/2007-00119.Tilg, PMID: 18235842 PMC2215762

[24] Yip WW Burt AD. Alcoholic liver disease. Semin Diagn Pathol. (2006) 23:149–60. doi: 10.1053/j.semdp.2006.11.002, PMID: 17355088

[25] Byrne CD Targher G. NAFLD: a multisystem disease. J Hepatol. (2015) 62:S47–64. doi: 10.1016/j.jhep.2014.12.012, PMID: 25920090

[26] Gao F Litchfield B Wu H. Adipose tissue lymphocytes and obesity. J Cardiovasc Aging. (2024) 4:5. doi: 10.20517/jca.2023.38, PMID: 38455510 PMC10919906

[27] Nishimura S Manabe I Nagai R. Adipose tissue inflammation in obesity and metabolic syndrome. Discov Med. (2009) 8:55–60. 19788868

[28] Bhatt S Guleria R Kabra S. Metabolic alterations and systemic inflammation in overweight/obese children with obstructive sleep apnea. PloS One. (2021) 16:e0252353. doi: 10.1371/journal.pone.0252353, PMID: 34086720 PMC8177414

[29] Mărginean C Meliț L Huțanu A Ghiga D Săsăran M. The adipokines and inflammatory status in the era of pediatric obesity. Cytokine. (2019) 126:154925. doi: 10.1016/j.cyto.2019.154925, PMID: 31759309

[30] Ullah A Singla R Batool Z Cao D Shen B. Pro- and anti-inflammatory cytokines are the game-changers in childhood obesity-associated metabolic disorders (diabetes and non-alcoholic fatty liver diseases). Rev endocrine Metab Disord. (2024) 25:783–803. doi: 10.1007/s11154-024-09884-y, PMID: 38709387

